# ASO Author Reflections: Transition to Robotic Radical Cholecystectomy: Building the Future on Strong Fundamentals

**DOI:** 10.1245/s10434-024-16075-1

**Published:** 2024-08-20

**Authors:** Gurudutt P. Varty, Shraddha Patkar, Mahesh Goel

**Affiliations:** Hepatobiliary and Retroperitoneal Sarcoma Division, Gastrointestinal and HPB Surgical Oncology, Tata Memorial Hospital, Homi Bhabha National Institute, Parel, Mumbai, India

## Past

Radical cholecystectomy is the recommended procedure for resectable gallbladder cancer (GBC), which entails en bloc removal of the gallbladder with a liver wedge/segment IVb and V resection and complete lymph nodal clearance of regional nodal stations (stations 8, 12 and 13). Traditionally, open radical cholecystectomy (ORC) is considered the gold standard in view of the complexity and oncological safety of the procedure. The proximity of these tumors to the porto-hilar structures with liver infiltration necessitates a core competence in complex portal dissections and liver resections ranging from a liver wedge to major hepatectomies, which may seem overwhelming even for hepatopancreatobiliary surgeons. Thus, the transition from open to radical cholecystectomy is a slow, arduous process with a steep learning and proficiency curve.

## Present

The safety and feasibility of robotic radical cholecystectomy (RRC) and comparable short-term outcomes to ORC in selected patients has already been established.^1–3^ Compared with laparoscopic surgery, the robotic platform provides a greater degree of freedom, maneuverability and precision. Thus, the open approach tends to transition better to a robotic platform rather than the laparoscopic approach, where all the steps of the ORC can be replicated. The RRC should emulate the same exposure and traction-countertraction techniques used in ORC as depicted in our video, for a safe and thorough oncological clearance.^4^ Thus, the most important tenet to achieve this technical equivalence in RRC is to master the technique of ORC.

## Future

At present, RRC is utilized for early GBCs with limited liver involvement and nodal burden. However, the limits of RRC will be pushed as we continue to evolve in managing GBCs along with the latest technology available at hand (intraoperative ultrasound, indocyanine green-guided resections and artificial intelligence). As the complexity of RRC increases, the need for proficiency in ORC cannot be overstated, in the event when intraoperative complications arise or the lack of progress calls for a conversion. Thus, the future of robotic surgery for GBC should be built on the strong fundamentals of ORC.
